# Activating transcription factor 4-dependent lactate dehydrogenase activation as a protective response to amyloid beta toxicity

**DOI:** 10.1093/braincomms/fcab053

**Published:** 2021-03-26

**Authors:** Teresa Niccoli, Fiona Kerr, Inge Snoeren, Daniel Fabian, Benjamin Aleyakpo, Dobril Ivanov, Oyinkan Sofola-Adesakin, Adam Cryar, Jennifer Adcott, Janet Thornton, Linda Partridge

**Affiliations:** 1 Department of Genetics, Evolution and Environment, Institute of Healthy Ageing, University College London, London WC1E 6BT, UK; 2 Department of Biological and Biomedical Sciences, School of Health & Life Sciences, Glasgow Caledonian University, Glasgow G4 0BA, UK; 3 Department of Life Sciences, School of Applied Sciences, Edinburgh Napier University, Edinburgh EH11 4BN, UK; 4 European Molecular Biology Laboratory, European Bioinformatics Institute, Wellcome Genome Campus, Hinxton, Cambridge CB10 1SD, UK; 5 UK Dementia Research Institute (UKDRI), Cardiff University, Cardiff CF24 4HQ, UK; 6 Max Planck Institute for Biology of Ageing, 50931 Cologne, Germany

**Keywords:** Alzheimer's disease, Drosophila, ATF4, Ldh, UPR

## Abstract

Accumulation of amyloid beta peptides is thought to initiate the pathogenesis of Alzheimer's disease. However, the precise mechanisms mediating their neurotoxicity are unclear. Our microarray analyses show that, in *Drosophila* models of amyloid beta 42 toxicity, genes involved in the unfolded protein response and metabolic processes are upregulated in brain. Comparison with the brain transcriptome of early-stage Alzheimer's patients revealed a common transcriptional signature, but with generally opposing directions of gene expression changes between flies and humans. Among these differentially regulated genes, lactate dehydrogenase (*Ldh*) was up-regulated by the greatest degree in amyloid beta 42 flies and the human orthologues (*LDHA* and *LDHB*) were down-regulated in patients. Functional analyses revealed that either over-expression or inhibition of *Ldh* by RNA interference (RNAi) slightly exacerbated climbing defects in both healthy and amyloid beta 42-induced *Drosophila*. This suggests that metabolic responses to lactate dehydrogenase must be finely-tuned, and that its observed upregulation following amyloid beta 42 production could potentially represent a compensatory protection to maintain pathway homeostasis in this model, with further manipulation leading to detrimental effects. The increased *Ldh* expression in amyloid beta 42 flies was regulated partially by unfolded protein response signalling, as *ATF4* RNAi diminished the transcriptional response and enhanced amyloid beta 42-induced climbing phenotypes. Further functional studies are required to determine whether *Ldh* upregulation provides compensatory neuroprotection against amyloid beta 42-induced loss of activating transcription factor 4 activity and endoplasmatic reticulum stress. Our study thus reveals dysregulation of lactate dehydrogenase signalling in *Drosophila* models and patients with Alzheimer's disease, which may lead to a detrimental loss of metabolic homeostasis. Importantly, we observed that down-regulation of *ATF4*-dependent endoplasmic reticulum-stress signalling in this context appears to prevent *Ldh* compensation and to exacerbate amyloid beta 42-dependent neuronal toxicity. Our findings, therefore, suggest caution in the use of therapeutic strategies focussed on down-regulation of this pathway for the treatment of Alzheimer's disease, since its natural response to the toxic peptide may induce beneficial neuroprotective effects.

## Teaser

Niccoli et al. report, in *Drosophila*, that neuronal lactate dehydrogenase, through activating transcription factor 4, is increased in response to Aβ42. Conversely, lactate dehydrogenase is reduced in inhibitory neurons, which are vulnerable to neurodegeneration in Alzheimer's disease patients. It is therefore possible that upregulation of Ldh maintains neuronal homeostasis.

## Graphical Abstract

**Graphical Abstract:**
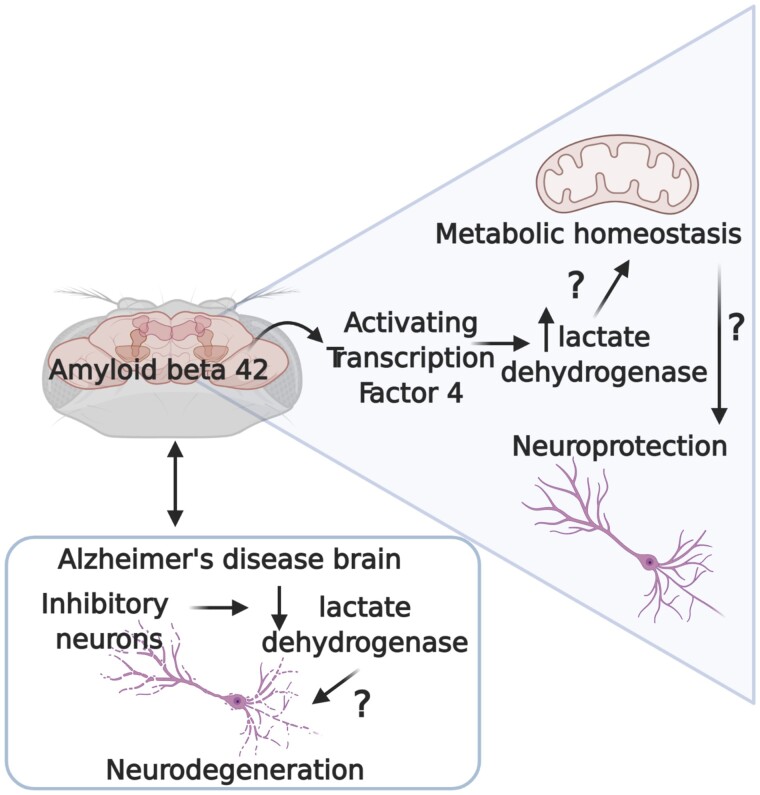

## Introduction

Alzheimer's disease is the most common form of dementia, and affects over 50 million people worldwide.^1^ The main risk factor for Alzheimer's disease is advancing age,^2^ with incidence increasing from 0.6% at age 60–65 to over 8% for people aged over 85.^3^ Although the age-specific incidence of Alzheimer's disease has declined in many parts of the world in recent years,^4^ human lifespan has increased steadily over the last decades.^5^ Alzheimer's disease is therefore becoming one of the most common causes of disability and death, with no effective preventative measures or cures yet available.

Alzheimer's disease is characterized by widespread neurodegeneration, but how this is mediated is still unclear. Pathologically, brains of Alzheimer's disease patients display an intracellular accumulation of neurofibrillary tangles, composed of Tau protein, and a substantial increase in extracellular amyloid plaques composed of amyloid beta (Aß) peptides, derived from the mis-processing of the amyloid precursor protein (APP). The most widely accepted model of Alzheimer's disease aetiology is the amyloid hypothesis, first postulated in 1992,^6^ and based on the observation that all early onset, dominantly inherited forms of the disease are caused by mutations that lead to the abnormal-processing of APP. The amyloid hypothesis states that Alzheimer's disease is initiated by the accumulation of toxic Aß peptides,^6^ which induce a downstream cascade of events, ultimately resulting in neuronal cell death. Yet, the mechanisms by which Aß accumulation leads to neuronal dysfunction remain to be resolved.

Efforts to identify pathways leading to neuronal cell death have been driven forward by recent advances in single-cell sequencing. This has facilitated the identification of cell-type-specific responses to accumulation of toxic entities and formulation of a more precise picture of the cellular phase of Alzheimer's disease pathogenesis,^7^ during which specific neuronal responses to the accumulation of toxic proteins ultimately lead to the demise of several neuronal populations. A recent study using single-cell sequencing of Alzheimer's disease patient brains has shown that different cell types show distinct and sometimes opposing transcriptional responses to disease.^8^ Whether these transcriptional events play a causal role in disease progression, or whether they reflect bystander responses to proteotoxicity, requires further study.

Model organisms play a key role in investigations where genes highlighted by human studies can be manipulated to determine whether they affect disease development.^9^*Drosophila* models are excellent for uncovering the molecular mechanisms of disease, thanks to their powerful genetic toolkit developed during the century that flies have been used in research.^10^ Moreover, 75% of human disease genes have homologues in flies.^11^ Additionally, their short lifespan enables the assessment of pathological responses to toxic insults across the lifespan of a complex organism. *Drosophila* Aß toxicity models are widely used in neurodegeneration research ^12^ and display a range of pathologies, including neuronal dysfunction, behavioural decline and early death in response to amyloid accumulation in the fly brain. We have used an inducible model to express pathogenic Arctic Aß42, tagged with an endoplasmic reticulum (ER) export signal peptide,^13^ specifically in neurons of the adult fly, thereby removing any confounding developmental effects. These flies show shortened lifespan, climbing defects and neurodegeneration phenotypes.^14^

Fly models have shown cellular responses to Aß accumulation similar to those seen in Alzheimer's disease patients. For example, the ER stress response is induced in fly models of Aß toxicity^15^ and in human Alzheimer's disease brain.^16^^,^^17^ Functional genomic approaches using *Drosophila* have confirmed that this is a protective response, since up-regulation of specific components of the Unfolded Protein Response (UPR), including Xbp1 and BiP,^15^^,^^18^ can protect against Aß toxicity. Similarly, Nrf2, a transcriptional activator of cell protection genes, has been shown to be altered in Alzheimer's disease patients,^19^ with some studies finding up-regulation and others down-regulation, possibly due to looking at different disease stages. Our studies in flies have shown that restoring Nrf2 activity can ameliorate Aß toxicity by promoting degradation of the amyloid peptide and increasing resistance to cellular stress.^20^ Therefore, *Drosophila* appear to mount an evolutionarily conserved response to Aß accumulation in the brain and, due to the ease of genetic manipulation, flies present a powerful tool to identify molecular mediators of Alzheimer's disease pathogenesis and thus potential targets for drug development and clinical translation.

We set out to identify potential age-specific responses to Aß accumulation in the *Drosophila* brain and to identify conserved transcriptional responses to amyloid toxicity between fly and human AD. Given that Alzheimer's disease is a late-onset disorder, we monitored transcriptional responses at different ages. However, the majority of transcriptional alterations in response to Aß42 in older fly brains overlapped with those in young flies, suggesting that these represent general brain-specific responses to amyloid early in disease pathogenesis. Comparing microarray analysis of fly brains to single-cell transcriptomics of brains of Alzheimer's disease patients, we identified several genes that are differentially expressed, although reciprocally regulated, in flies and humans, including genes implicated in metabolism, ER-stress, proteostasis and cell cycle. We further identified *Ldh* as a conserved gene mis-expressed in the presence of Aß downstream of ATF4-dependent UPR activation. Activation of the UPR could potentially modulate a protective metabolic response in Alzheimer's disease that warrants further investigation for potential therapeutic benefit.

## Materials and methods

### *Drosophila* microarray analyses

Upstream Activating Sequence *UAS-ArcAß42/+; elavGS/+* flies were treated, for 7 days, with standard SY medium containing 200 µM Mifepristone (RU486) or medium containing carrier alone (−RU) from either 5 or 20 days of age and brains dissected one week after withdrawal of induction, at 19 or 34 days of age, respectively. Treatments for each replicate were staggered and brains dissected on consecutive days over a 2-hour period to circumvent circadian effects. Frozen fly heads from each replicate were used to measure Aß42 peptide concentrations by enzyme-linked immunosorbent assay (ELISA).

Brain tissues used for microarray analyses were stored in Allprotect tissue reagent (Qiagen # 76405) at −80°C and, for each array, RNA extracted from 25 brains using RLT buffer + 0.01% β-mercaptoethanol and purified with RNeasy columns (Qiagen, Valencia, CA, USA) following the manufacturer's instructions. The quality and concentration of RNA was confirmed using an Agilent Bioanalyzer 2100 (Agilent Technologies, Santa Clara, CA, USA), and further procedures followed the standard Affymetrix protocol. All samples were hybridized to the *Drosophila* Genome 2.0 Genechip. In total, 5 biological replicates of each condition (−RU 19d, +RU [d 5–12] 19d, −RU 34d and +RU [d 20–27] 34d) were performed.

#### Differential expression analysis of *Drosophila* brain data-sets

Differential gene expression was determined as previously described.^20^ Briefly, raw data (cel files) were processed to correct for probe-sequence biases using gcrma (http://www.bioconductor.org) in R (http://www.r-project.org) and presence of target transcripts, with a *P*-value <0.111, determined using Affymetrix's MicroArray Suite 5.0 (bioconductor's package affy).^21^ Raw data were summarized and normalized using the Robust Multichip Average (rma) function (bioconductor's package affy^22^). A linear model was fitted and differential expression of genes was assessed using the empirical Bayes moderated *t*-statistic in R's limma package.^23^*P*-values were adjusted for multiple hypothesis testing by applying the Benjamini and Hochberg correction for false discovery rate. Summarized probe-sets were mapped to transcripts using R's package ‘drosophila2.db’. Transcripts not mapping to any known or predicted genes were excluded from further analysis.

#### Gene ontology analysis of *Drosophila* brain data-sets

Pathway analyses were performed as previously described.^20^ The Wilcoxon rank sum test, as implemented in Catmap,^24^ was used to determine significant enrichment of Gene Ontology (GO) categories. FlyBase (http://flybase.org) gene identifiers were mapped to Gene Ontology identifiers (FlyBase version FB2014_01). Ranks of genes were based on the *P*-value derived from the Bayes *t*-statistic for differential expression. To account for multiple hypothesis testing, an enrichment of GO terms was deemed statistically significant if the *P*-value derived from the Wilcoxon rank sum test was ≤1.0 × 10^−^^05^. Gene lists were sorted by log-fold change and *P*-value. For all microarray experiments, two sets of lists were derived; a gene list comprising most differentially up-regulated (log-fold change > 0) genes at the top of the list and most differentially down-regulated genes (log-fold change < 0) at the bottom of the list (termed up-to-down) and vice versa (termed down-to-up). If a GO category was found to be statistically significant in the up-to-down list, this GO was referred to as up-regulated, and conversely if statistically significant in the down-to-up list, this GO was referred to as down-regulated, meaning that a large enough proportion of genes in these categories were found to be up or down-regulated respectively. Statistical significance of overlaps of GOs between age-groups was determined using Fisher's exact test. To account for multiple hypothesis testing, a *P*-value cut-off of ≤1.0 × 10^−^^05^ was used.

#### Comparison of human Alzheimer’s disease single-cell transcriptional changes versus *Drosophila* models

Human genes differentially expressed between early and no Alzheimer pathology in six cell types were obtained from Supplementary Table 2 of Mathys et al. Human genes were considered significant according to the author’s definition (column ‘DEGs.Ind.Model’). Fly genes were defined as differentially expressed if their adjusted *P*-value was <0.05. To analyse sharing in candidate genes between *Drosophila* and humans, we first transformed human candidate genes to fly orthologues using a table with human to *Drosophila* orthologue mappings from the Alliance of Genome Resources (AIG) (Alliance of Genome Resources), which employs the *Drosophila* RNAi Screening Center Integrative Ortholog Prediction Tool. Only genes for which the forward (human to fly orthologues) and reverse orthologue search (fly orthologues to human genes) resulted in the same top gene hits were included (i.e. columns ‘BestForward’ and ‘BestReverse’ were filtered for ‘yes’). We included all matching orthologue hits when one human gene mapped to multiple fly orthologues. Where different human genes mapped to the same fly orthologue, the orthologue hit was only considered once. Genes that were not in common between the above *Drosophila* brain and human single-cell datasets were removed from the analysis, so that the background size varied between 4,464 and 5,757 genes dependent on the human cell type. Shared candidate genes between the *Drosophila* and human cell type datasets were then obtained and SuperExactTest (Wang et al.) used to assess whether the number of overlapping genes is significantly different than expected by chance. Performing the analysis based on human genes resulted in qualitatively similar results (not shown).

### Fly husbandry and stocks

All flies were reared at 25°C on a 12-h:12-h light:dark cycle at constant humidity and on standard sugar-yeast-agar (SYA) medium (agar, 15 g/l; sugar, 50 g/l; autolyzed yeast, 100 g/l; nipagin, 30 ml/l (of 10% solution in ethanol) and propionic acid, 2 ml/l). For induction with RU486, 24–48 h after eclosion, the female flies carrying a heterozygous copy of *elavGS* and at least one *UAS* construct were fed SYA medium supplemented with 200 µM mifepristone (RU486) to induce transgene expression. *ElavGS*, derived from the original *elavGS 301.2 *line^25^ was a gift from Dr H. Tricoire (CNRS), the *UAS-Aß42Arc* stock was a gift from Dr D. Crowther (University of Cambridge). The *UAS-Ldh stock* was generated by PCR amplifying the genomic locus with primers (ATGGCCGCCATTAAGGACAGTCTGTTGGC and TTAGAACTTCAGACCAGCCTGGACATCGGA), and gateway cloned into an entry vector and transferred into a gateway compatible *pUASTattB* vector according to standard protocols and inserted into the attP40 locus. *LdhRNAi* stock: *y1 v1; P{TRiP.HMS00039}attP2, P{lacW}simaj11B7* and *ATF4 RNAi* stock, *P{TRiP.JF02007}attP2* were all from Bloomington stock centre. All flies were back-crossed six times into a *w1118* (for over-expression lines) or a *v1w+* background (for RNAi lines) line to ensure homogenous back-ground.

### Lifespan analysis

Flies were raised at a uniform density in 200 ml bottles. After eclosion, flies were allowed to mate for 24–48 h. At least 110–150 females of the appropriate genotype were split into groups of 15 and housed in vials containing SYA medium and either carrier alone or RU486. Deaths were scored and flies tipped onto fresh food three times a week. All lifespans were performed at 25°C.

### Climbing assay

The climbing assays for Aß expressing flies were performed as previously described.^14^ Briefly, 15 flies were placed in a 25 cm pipette, tapped to the bottom, and allowed to climb for 45 s. The number of flies in the top 5 cm, centre, and bottom 3 cm was scored. A performance index was calculated for each time point and plotted. For flies expressing *Ldh* and *LdhRNAi* in neurons climbing assays were performed according to Woodling et al.^26^ Briefly, flies allowed to climb in a vertical 20 cm column formed by two plastic vials, each fly height was scored in ImageJ and used for statistical analysis. Climbing assays were performed every 3–4 days and at least 100 flies were used per condition.

#### Quantitative PCR

Total RNA was extracted from 15 to 20 fly heads using Trizol (Invitrogen) and subsequently treated with DNAse I (Ambion) for DNA digestion. The RNA was then reverse transcribed using Superscript II (Invitrogen) with oligo(dT) primers. Quantitative gene expression analysis was performed on a 7900HT real-time PCR system (Applied Biosystems) using SYBR-green technology (ABI). Relative quantities of transcripts were determined using the relative standard curve method normalized to *eIF*. Primer sequences can be found in Table 1.

**Table 1:** Primers for quantitative real-time PCR analysis

Gene	Forward Primer	Reversed primer
Aß	CGATCCTTCTCCTGCTAACC	CACCATCAAGCCAATAATCG
Atf-4	TCGATGCTTACAAACAGGCG	AAAGTTAAAGGGCGTGGCAG
eIF	ATCAGCTCCGAGGAT	GCGGAGACAGACGTT
LDH	GGTATCGGGACTGTA	GCAGCACGGCTCCAACTTTC
Sima	CACCTTCAAGAGCGTGCTGA	CGTGGCCTGGCTAAGAATC

#### LDH assay

To measure LDH activity, 10 fly heads were homogenized in 62.5 µl of 0.2 M NaPO_4_, pH 6.5 plus 0.2% phenylthiourea. The samples were centrifuged at 21,000 *g* for 5 min at 4°C, the clear supernatant was taken to measure the activity of LDH. Protein extracts were quantified using the Bradford protein assay (Bio-Rad protein assay reagent; Bio-Rad laboratories Ltd (UK)). For the measurement in the direction pyruvate to lactate, the assay mixture contained 0.05 M sodium phosphate, pH 6.5, 1 mM sodium pyruvate, and 0.2 mM NADH. For the measurement in the direction lactate to pyruvate, the assay mixture contained 0.1 M sodium phosphate, pH 7.5, 100 mM D, L-lithium lactate, and 4.13 mM NAD+. Blank samples contained everything but NADH or NAD+. 10 µg of the protein extracts were added to the reaction mixture. The activity of LDH was measured spectrophotometrically at 25°C at 340 nm on a Tecan Infinite M200 platereader. The LDH activity is represented as a slope of the reaction.

### Lactate pyruvate levels

To measure lactate and pyruvate levels, 15 fly heads were homogenized in 30 µl 4% cold Trichloroacetic acid. The samples were centrifuged for 15 min at 11,500 *g* for 15 min at 4°C. 20 µl of the clear supernatant was neutralized with 180 µl of 1:10 dilution of 1 M Tris-HCL pH8. Lactate and pyruvate levels were measured using the Lactate assay kit (Sigma) and the Pyruvate assay kit (Sigma). The samples were diluted 1:10 in the assay buffer, then the pyruvate and lactate levels were performed according to the manufacturers’ instructions. The pellet was resuspended in 75 µl of 10 mM Tris pH 10.4 to extract the proteins. Protein extracts were quantified using the Bradford protein assay (Bio-Rad protein assay reagent; Bio-Rad laboratories Ltd (UK)). The amount of lactate and pyruvate in each sample is expressed as the ratio of the total protein content (µg/g total protein).

#### Aß42 ELISA

Total Aß42 was extracted from fly heads in GnHCl buffer (5 M Guanidinium HCl, 50 mM Hepes pH 7.3, protease inhibitor cocktail (Sigma, P8340) and 5 mM EDTA), as previously described (Sofola O et al.). Aß42 levels were then measured using the High Sensitivity Human Amyloid Aß42 ELISA kit (Millipore), according to the manufacturers’ instructions. Protein extracts were quantified using the Bradford protein assay (Bio-Rad laboratories Ltd, UK) and the amount of Aß42 in each sample expressed as a ratio of the total protein content (pmoles/g total protein).

### Fluorescence-activated cell sorting of neurons

Green Fluorescent Protein (GFP) labelled neurons were sorted according to DeSalvo et al.^27^ Briefly, 10 brains per sample were dissected in Schneider’s medium containing 1% Bovine Serum Albumin and quickly transferred into the same medium on ice. Samples were dissociated as described previously and GFP positive neurons were fluorescence-activated cell sorting (FACS) sorted straight into lysis medium, ready for RNA extraction.

### Statistical analyses

Microarray analyses are described above. For climbing assays, data were analysed by ordinal logistic regression or linear regression in R (http://www.r-project.org), using the individual heights for each fly as data points. For lifespans, data are presented as cumulative survival curves and survival rates were compared using log-rank tests in Excel. For all other experiments, data are presented as means ± SEM, from a minimum of 3 independent biological repeats, and were analysed by unpaired student’s *t*-test or one-way ANOVA and Tukey’s post-hoc analyses using Graphpad Prism 8.0 (https://www.graphpad.com/scientific-software/prism/).

### Data availability

The raw microarray data generated in this study are deposited in ArrayExpress (http://www.ebi.ac.uk/arrayexpress) with identifier E-MTAB-8865.

## Results

**Figure 1:**
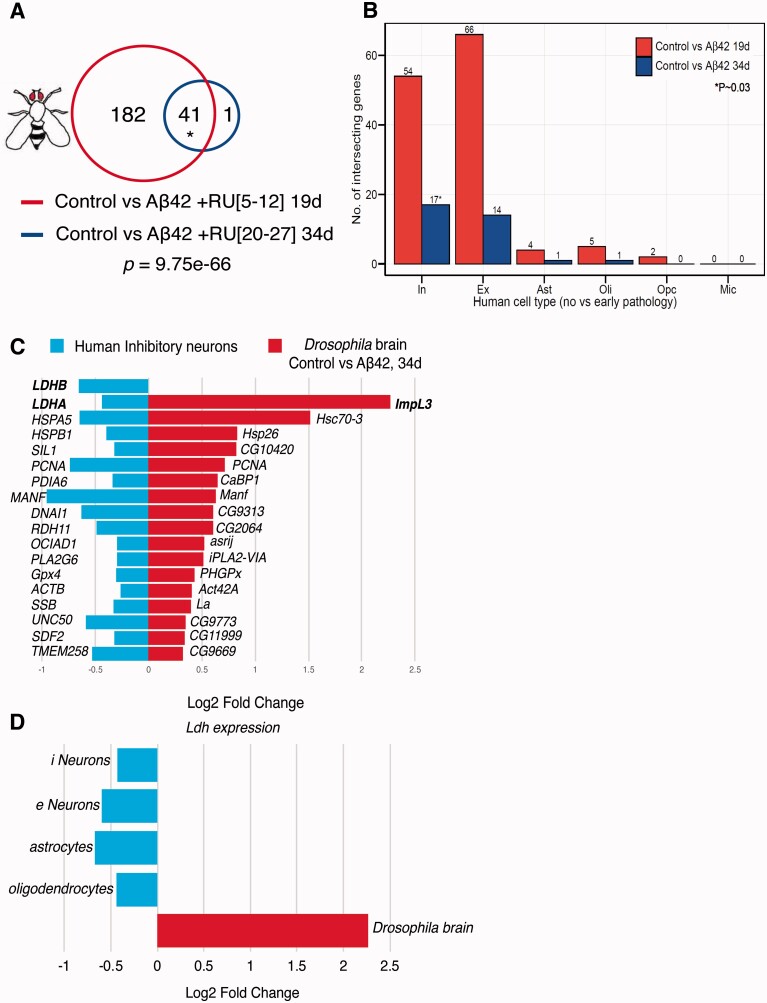
**Overlapping transcriptional responses to early AD pathology in aged *Drosophila a*nd human brain.** (**A**) Significant overlaps in differentially expressed (DE) genes were observed between young (19d) and old (34d) fly brains in response to Aß42 expression (41 of 224 DE genes analysed; *p *=* *9.75e-66, Bayes moderated *t*-statistic and Benjamini and Hochberg FDR correction), and individual genes are detailed in Supplementary Fig. 1C. (total gene number *n* = 8566). (**B**) Plot depicts the number of intersecting genes that are differentially expressed in no versus early pathology in human AD brain, across various cell types, and control versus Aß42 expression in fly brain. Red represents overlaps between human genes and alterations in young flies and blue overlaps with old flies. Significant overlaps (*P* = 0.03; SuperExactTest) were observed only when comparing no versus early pathology in human inhibitory neurons and control versus Aß42+ 34d (old flies). Abbreviations: In, inhibitory neurons; Ex, excitatory neurons; Ast, astrocytes; Oli, oligodendrocytes; Opc, oligodendrocyte precursor cell; Mic, microglia. (**C**) Plot showing the 17 genes from the significant overlap in **B** (*). Genes are upregulated in flies (red) and downregulated in humans (blue). (**D**) *Ldh* was the principal upregulated gene in response to Aß42 in aged *Drosophila* brain and its human orthologue *LDH* was significantly downregulated in inhibitory (i) neurons (LDH A&B). *LDHB* expression was also downregulated in human neurons excitatory (e), astrocytes and oligodendrocytes in brain tissue from early-stage AD patients compared with controls. The fly image in this figure was originally created by Dr Fiona Kerr and produced in Kerr et al.^56^

**Figure 2:**
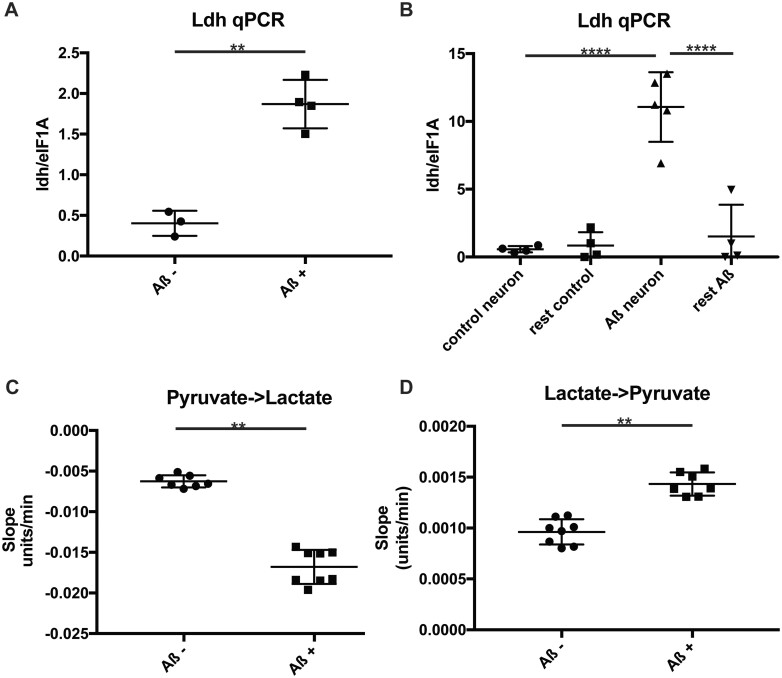
**Ldh is upregulated in Aß expressing fly brains.** (**A**) Ldh qPCR analysis of brains expressing Aß (Aß+) and uninduced controls (Aß−). Genotypes: UAS-Aß; elav GS. (**B**) Ldh qPCR analysis of FACS sorted GFP expressing neurons and other cells, expressing Aß (Aß) and driver alone (controls). Genotypes: *UAS-Aß/UAS-eGFP; elav GS* and *UAS-eGFP; elav GS.* (**C and D**) Ldh enzymatic assay on brain extracts expressing Aß (Aß+) and uninduced controls (Aß−). Assayed in the direction of lactate (**C**) and pyruvate production (**D**). Values shown are the slopes generated by the enzymatic reaction. Lactate production generates a negative slope, so a lower negative value signifies a greater activity. Genotypes: *UAS-Aß; elav GS.* **A, C** and **D** were compared by *t*-test, **B** by one-way ANOVA followed by Tukey’s post-hoc test. ** *P *<* *0.01, ****P <* *0.0001, *N* = 3–5 per condition for qPCR; *N* = 7–8 per condition for LDH activity.

To understand how Aß toxicity is mediated in neurons, and whether age influences this response, we measured RNA expression in the brains of flies expressing Aß42 peptide either early or later in life (Supplementary Fig. 1A and B). The lifespan curves associated with these induction patterns have been published elsewhere (Fig. 2C in the study by Rogers et al.^28^) 224 genes were differentially expressed between uninduced controls and Aß42-expressing brains at the two ages (Supplementary Tables 1 and 2), 41 of which were in common between the young and older flies, a significant enrichment (Fig. 1A, Supplementary Fig. 1C and Table 3). A larger number of genes were altered in response to Aß42 in young flies (Fig. 1A), possibly because young flies can activate a more robust response, which might also explain why older individuals are more susceptible to Aß toxicity. Most genes with altered expression at older age were also altered in the young brains, suggesting that the majority of these represented generic responses to Aß42, albeit with magnitudes that varied slightly with age (Fig. 1A, Supplementary Fig. 1C).

**Figure 3:**
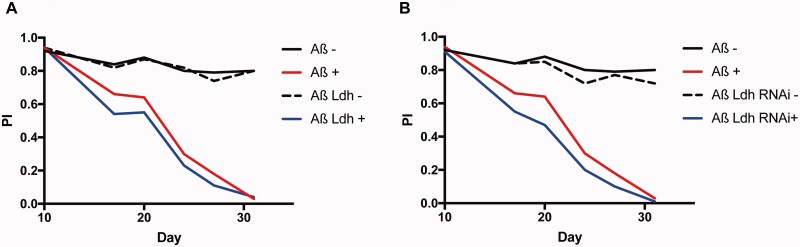
**Manipulation of Ldh leads to exacerbation of climbing defects.** (**A**) Plot of Performance index for a negative geotaxis assay of flies expressing Aß (Aß+) alone compared to flies expressing Aß and Ldh (Aß Ldh+), and their un-induced controls (Aß− and Aß Ldh−). Genotypes: *UAS-Aß/UAS-Ldh; elav GS, UAS-Aß; elavGS. Aß UAS Ldh* was not significantly different to Aß alone (*P *=* *0.139 by ordinal logistics regression). (**B**) Plot of Performance index for a climbing assay of flies expressing Aß (Aß+) alone compared to flies expressing Aß and Ldh RNAi (Aß Ldh RNAi +), and their un-induced controls (Aß− and Aß Ldh RNAi−). Genotypes: *UAS-Aß/UAS-Ldh RNAi; elav GS, UAS-Aß; elavGS. Aß Ldh RNAi* genotype was significantly worse than Aß alone (*P* = 0.00552 by ordinal logistics regression). Note experiments were run in parallel with Aß+ and Aß− curves in Fig. 3a, but depicted separately for clarity.

A recent single-cell study also found that transcriptional changes in Alzheimer’s disease patients early and later in disease development were quite similar, suggesting that there is a strong transcriptional signature early in response to Aß42 that does not greatly change with disease progression.^8^ To determine if the changes in gene expression in *Drosophila* brains were conserved in human Alzheimer's disease brains, we compared our young and old fly data-sets to single-cell sequencing of early and late stage Alzheimer's disease patients versus healthy controls^8^ (Fig. 1B, Supplementary Table 8). Significant overlaps were observed only between control versus Aß42 34d in flies and no pathology versus early Alzheimer's disease pathology in inhibitory neurons in humans (Fig. 1B), with 17 genes commonly regulated between Alzheimer's disease flies and patients (Fig. 1C). Of these, *lactate dehydrogenase* (*Ldh*; *Drosophila ImpL3*) was up-regulated to the greatest degree in Aß42 fly brains (Fig. 1C and D), while both *LDHA* and *LDHB* isoforms were down-regulated in inhibitory patient neurons. *LDHB* was also significantly down-regulated in excitatory neurons, astrocytes and oligodendrocytes of Alzheimer's disease patients with early pathology (Fig. 1D).

Moreover, several genes involved in the Unfolded Protein Response (UPR) including *Hsc70, CG10420* and *CaBP1* were significantly up-regulated in Aß42 fly brain, and their orthologues *HSPA5/BiP, SIL1* and *PDIA6* down-regulated in inhibitory neurons of Alzheimer's disease patients (Fig. 1C), consistent with ER-stress associated responses under Alzheimer's disease conditions in flies and humans. Supporting this observation, GO pathway analyses in *Drosophila* (Supplementary Tables 4–7) confirmed that UPR, ER and Golgi processes were significantly enriched in differentially expressed upregulated genes in both young and old Aß42 fly brain (Supplementary Tables 4 and 6). These data suggest that our fly model represents early stages of Alzheimer's disease pathogenesis, but with mainly opposing effects on expression of the same genes, including defects in LDH and UPR levels. This discrepancy in the direction of change requires further investigation, but may represent cell-type specific effects which are not detectable using a whole-brain approach to transcriptional analyses in flies compared to human studies.

LDH catalyses the conversion of lactate into pyruvate and vice-versa and is a key enzyme in the glycolytic cascade. Its activity is increased in Alzheimer's disease patients' brains,^29^ and mRNA levels of *LDHA* are higher in fibroblasts derived from late-onset Alzheimer's disease patients versus controls.^30^ However, it is unclear whether LDH plays a direct role in Alzheimer's disease pathogenesis. Up-regulation of glycolysis in neurons, and specifically of key enzymes, including LDHA, confers resistance to Aß toxicity.^31^ We, therefore, sought to understand whether the increase in LDH activity observed in flies is itself a compensatory response to Aß42, or whether instead it contributes to disease development.

We confirmed by qPCR that expression of Aß in fly neurons leads to up-regulation of *Ldh* RNA in fly brains (Fig. 2A), and this upregulation does not occur in driver alone flies treated with RU (Supplementary Fig. 2A). This is accompanied by increased enzymatic activity in both directions (Fig. 2C and D). However, this increase in enzymatic activity did not result in an increase in either lactate or pyruvate (Supplementary Fig. 2B and C). This was, possibly, because flux through the pathway was increased in both directions, and the steady-state metabolite concentrations were hence maintained. Ldh is expressed both in neurons and in glia, where it contributes to the neuronal lactate shuttle. In glia, Ldh catalyses the conversion of pyruvate to lactate, which is then shuttled to neurons via monocarboxylate transporters where Ldh converts it back to pyruvate which then enters glycolysis for rapid energy production.^32^ Whereas in mammalian systems these two reactions are catalysed by different isoenzymes, composed of different subunits, in flies there is a single *Ldh* gene (*Impl3*), with the same enzyme catalysing the reaction in both directions. We performed the above enzymatic analysis of Ldh using whole head lysates, hence further studies were required to determine whether the increase in LDH activity occurred in neurons or in glia.

To identify which cell-types were responsible for the increase in LDH activity in Alzheimer's disease flies, we next measured transcript levels in isolated neurons versus non-neuronal cells in the fly. We FACS sorted GFP positive cells, using control or Aß42 flies co-expressing GFP specifically in neurons. We confirmed that the GFP sorted cell fractions were indeed enriched for neurons by assessing the levels of the neuronal marker elav (Supplementary Fig. 2D). We then measured *Ldh* mRNA levels on FACS sorted cells, and found that *Ldh* increased in neurons (Fig. 2B) but not in the other cell types in the brain.

To test whether an increase in Ldh was protective, we generated flies over-expressing *Ldh* in adult neurons and confirmed the over-expression (Supplementary Fig. 3).

We then over-expressed *Ldh* in adult neurons that also expressed Aß42, and found an exacerbation of the impaired climbing phenotype observed in these flies, suggesting that *Ldh* up-regulation may contribute to Aß42-induced neuronal toxicity (Fig. 3A). qPCR analysis of *Ldh* transcripts showed that, even in the −RU condition, the *UAS-Aß; UAS-Ldh* fly lines showed increased *Ldh* expression (Supplementary Fig. 3A), leading to increased Ldh enzymatic (Supplementary Fig. 3B and C). The increase in transcript and enzyme activity in the UAS line in the un-induced condition most likely reflects the leakiness of the elavGS driver.^33^ There was a trend towards increased lactate levels (which did not reach significance) and there was a slight decrease in pyruvate in the over-expression line (Supplementary Fig. 3D and E), possibly indicating that excessive Ldh activity in neurons is driving a higher rate of pyruvate consumption by the TCA cycle.

To test this further, we down-regulated *Ldh* using RNAi, in flies over-expressing Aß42, but this also led to a worsening of the climbing phenotype (Fig. 3B). Again, qPCR analysis, showed that *Ldh* transcripts were down-regulated, even in the non-induced conditions, leading to changes in enzymatic activity and metabolite levels both in the induced and un-induced condition. This is consistent with previous studies showing that RNAi lines can display strong knock-down even in the un-induced condition.^33^ Pyruvate and lactate levels were very low in the presence of Ldh RNAi, however, suggesting that Ldh enzyme is affecting the production of both metabolites under our experimental conditions (Supplementary Fig. 3), possibly because of a compensatory down-regulation of the whole pathway, but this remains to be determined.

Over-expression or knock-down of *Ldh*, however, also negatively affected climbing behaviour in healthy flies (Supplementary Fig. 3F and G) suggesting that its expression must be finely-controlled to maintain metabolic homeostasis and neuronal function. As further genetic manipulation of *Ldh* also induced detrimental effects in Aß42-expressing flies, these findings could indicate that its natural up-regulation in response to Aß42 production represents a compensatory mechanism to maintain optimal levels of gene expression and protection against metabolic dysfunction under these conditions.

**Figure 4:**
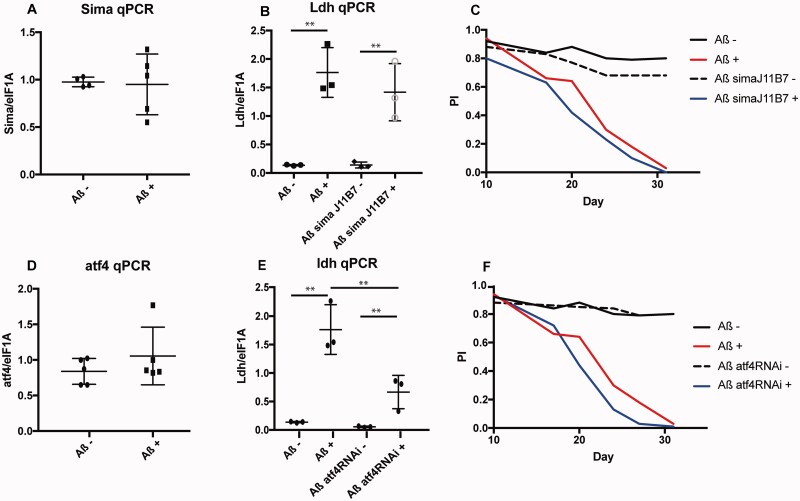
**Sima does not but ATF4 does induce *Ldh*.** (**A**) qPCR for *Sima* in fly heads expressing Aß (Aß+) and un-induced controls (Aß−). Genotype: *UAS-Aß; elav GS*. (**B**) qPCR of *Ldh* in heads from *sima* mutant flies expressing Aß (*Aß sima J11B7*+) and their un-induced controls (Aß−, Aß sima J11B7−) Genotypes: *UAS-Aß; elav GS/sima J11B7, UAS-Aß; elavGS*. **P <* *0.01, *N* = 3–5 per condition. (**C**) Plot of Performance index for a climbing assay of flies expressing Aß alone (Aß+), together with mutant sima (Aß sima J11B7+) as well as their uninduced controls (Aß−, Aß sima J11B7−). Genotypes as above. *UAS-Aß/sima J11B7* genotypes were significantly worse than *Aß* alone (*p *=* *4.03e-06 respectively by ordinal logistics regression) but there was no interaction with RU, suggesting the *sima* mutation affects control and Aß expressing flies similarly. Note experiments were run in parallel with Aß+ and Aß− curves in Fig. 3a, but depicted separately for clarity. (**D**) qPCR for ATF4 in fly heads expressing Aß (Aß+) and un-induced controls (Aß−). Genotype: *UAS-Aß; elav GS*. (**E**) qPCR for *Ldh* in heads from flies expressing Aß together with RNAi for *ATF4.* Genotypes: *UAS-Aß; elavGS/UAS-ATF4 RNAi and UAS-Aß; elav GS.* **P <* *0.01, *N* = 3–5 per condition. (**F**) Plot of Performance index for a climbing assay of flies expressing Aß alone (Aß+), together with RNAi for *ATF4* (Aß ATF4RNAi +) and their uninduced controls (Aß− and Aß ATF4RNAi−). Genotypes as above. The *Aß ATF4RNAi* genotype displayed a significantly worse response to RU over time relative to Aß alone (*P *=* *0.03379 for a three-way interaction of RU, genotype and day by ordinal logistics regression). Note experiments were run in parallel with Aß+ and Aß− curves in Fig. 3a, but depicted separately for clarity.

We next investigated the potential molecular mechanisms responsible for regulation of *Ldh* in response to Aß42. *Ldh* levels are regulated by hypoxia inducible factor 1 (HIF1)^34^^,^^35^ in mammalian systems and by the *Hif1* homologue, *similar (sima)*, in flies.^36^ In Aß-resistant human neurons, elevation of Hif1 levels is thought to lead to increased *LDHA*^31^^,^^37^ and Hif1 protein levels are increased in Alzheimer's disease mouse models.^31^ To investigate whether sima was responsible for the up-regulation of *Ldh* in Alzheimer's disease fly brain in response to Aß, we first examined the levels of *sima* transcript, and observed no significant difference. However, *sima* can be regulated post-transcriptionally, so this does not rule out its involvement. We down-regulated *sima* genetically, using a heterozygous null mutant,^38^ but this had no effect on *Ldh* expression in brains of Aß over-expressing flies (Fig. 4B), suggesting that sima does not regulate *Ldh* levels in response to Aß42. Down-regulation of *sima* exacerbated the negative geotaxis phenotype of both Aß and control flies, suggesting that it is generally detrimental to fly climbing but not specifically to Aß toxicity.

ATF4 is an effector of the UPR, induced downstream of protein kinase R-like endoplasmic reticulum kinase (PERK) and eukaryotic Initiation Factor 2 alpha (eIF2alpha),^39^ and has been shown to regulate glucose homeostasis and energy expenditure.^40^ In particular, in flies it has been shown to up-regulate glycolytic enzymes, including Ldh in response to ER stress.^41^ The UPR is up-regulated in response to Aß in patients,^42^ animal models^43^ and flies.^18^*ATF4* is also induced in Alzheimer's disease patient brains^44^ and in animal models of Alzheimer's disease.^45^ Given that both *Ldh* and ER-stress associated genes were altered in response to Aß42 in our flies, we therefore further explored potential connections between these processes by assessing *ATF4* level and its potential functional role in mediating neuronal damage in AD. *ATF4* transcript was unaltered in Aß42 expressing flies compared to uninduced controls (Fig. 4D). However, as ATF4 is translationally regulated,^39^ alterations in mRNA may not be expected. Indeed, down-regulation of *ATF4* by RNAi dampened the increased expression of *Ldh* in response to Aß42 (Fig. 4E), suggesting that ATF4 does contribute, at least partially, to the regulation of *Ldh* under these conditions. Aß transcripts, on the other hand, were unaltered (Supplementary Fig. 4), suggesting the effect is specific to *Ldh*. *ATF4* down-regulation further decreased the climbing ability of Aß expressing flies (Fig. 4F), providing correlative evidence to suggest that activation of ATF4 could contribute to a protective response to Aß42 accumulation, possibly via its up-regulation of *Ldh*. More experiments will be required to prove this is the case.

## Discussion

We have shown that flies mount a conserved transcriptional response to the accumulation of Aß42, and that this response is similar in young and old flies. Similarly, in humans, it was found that the ageing signature was orthogonal to the disease response in Alzheimer's disease patients.^8^ Our findings highlight *Ldh*, as well as several ER-stress associated genes, as a major transcriptional responder to early Aß42 toxicity in both young and old fly brain. Greater transcriptional responses were detected in response to Aß42 in the brain of young flies, but further studies are required to confirm whether these represent protective responses that are lost with age.

Consistent with our observation that both *Ldh* and ER-associated genes are altered in both Aß fly models and Alzheimer's disease patient brain, we have also shown that expression of Aß42 induces *Ldh* expression, and subsequent activity, in fly brain via ATF4, a downstream effector of the UPR. The induction of Ldh could potentially be neuroprotective since both down-regulating *Ldh* and blocking the Aß42-induced increase in *Ldh* expression using *ATF4* RNAi in neurons are detrimental. Up-regulation of Ldh has been observed in Alzheimer's disease patients' brains.^29^ Elevated CSF Ldh activity is also used as an indicator of neuronal damage,^46^ although the exact source of this increase is not clear^46^ and could be due to both cellular and blood–brain barrier damage releasing the enzyme into the CSF or a direct increase in enzymatic activity of CSF-expressed Ldh. Our findings in the fly, are consistent with other studies showing that up-regulation of Ldh^37^ and other glycolytic enzymes^31^ increases resistance to Aß toxicity in cortical neurons, however, further experiments will be required to directly prove this, for example by blocking the ATF4 induced increase in Ldh and checking whether this has a detrimental effect.

We show for the first time that the Aß42 peptide directly induces *Ldh* expression in an animal model.

It is interesting that the fly response appears reciprocal to the one discovered in inhibitory human neurons.^47^ Inhibitory neurons are extremely susceptible to Aß toxicity. Severe loss of inhibitory GABAergic neurons has also been observed in several animal models of Alzheimer's disease and in patients,^47^^,^^48^ where their degeneration appears to precede that in other cell types, at least in early disease stages.^49^ It is possible, therefore, that fly neurons can mount a protective response to Aß, whereas inhibitory neurons display a loss of protective pathways, thus making them selectively susceptible to disease. Alternatively, by measuring transcriptional responses in heterogeneous neuronal and glial cell types using a whole-brain approach, our study may have excluded detection of cell-type specific transcriptional changes in our fly model. Hence, further work is required to investigate these functional connections using human neuronal models of Aβ toxicity.

*LDH* upregulation in mammalian systems has been shown to be regulated by a variety of mechanisms,^46^ one of the most prevalent being Hif1. Hif1 is responsible for the up-regulation of glycolysis in cancer cells, leading to the switch from oxidative to glycolytic metabolism, through the Warburg effect, which promotes their survival.^46^ It has been proposed that survival of neurons in Alzheimer's disease can also be promoted by the Warburg effect,^37^ and that this is also mediated by Hif1. However, in our *in vivo* model of Aß42 toxicity the transcription factor ATF4, and not sima (the fly Hif1 homologue), is responsible for the induction of *Ldh* expression in response to Aß42.

These findings suggest that Ldh upregulation is downstream of UPR activation. The UPR is mediated by 3 effectors: PERK, Iris and Atf6.^50^ PERK phosphorylates eIF2alpha to inhibit canonical translation and induce the translation of specific factors, such as ATF4. The UPR is induced in the brain of Alzheimer's disease patients and in animal models,^51^ however, whether this is pathological or protective is controversial.^51^ In particular, up-regulation of ATF4 has been observed in Alzheimer’s disease patients' brains,^44^ downstream of PERK activation and eIF2alpha phosphorylation.^44^ The phosphorylation of eIF2alpha is usually considered pathological in neurodegenerative conditions^52–54^ and its pharmacological or genetic inhibition has been shown to protect animal models of frontotemporal dementia^55^ and Alzheimer's disease.^54^ Functionally, however, its role in early pathogenesis of Alzheimer's disease is far from clear, with studies in cells suggesting that the up-regulation of eIF2alpha phosphorylation and ATF4 activation contributes to cellular resistance to Aß toxicity.^44^ Our *in vivo* data using a fly model of Aß42 toxicity agree with this finding and further suggest that ATF4 might also play a protective role, potentially by contributing to *Ldh* induction. However, formally demonstrating that this is indeed the case would require deleting the binding sites for ATF4 in the *Ldh* endogenous promoter and showing that this abrogates Ldh induction resulting in a detrimental effect in the presence of Aß.

This work requires further investigation, however, it lends a word of caution towards therapies geared purely towards down-regulating the UPR as a therapy for Alzheimer's disease, since part of its endogenous response may be beneficial.

## Supplementary material

Supplementary material is available at *Brain Communications* online.

## Supplementary Material

fcab053_Supplementary_Data

## Abbreviations

Aß =: amyloid ß
AD =: Alzheimer's disease
APP =: amyloid precursor protein
ATF4 =: activating transcription factor 4
Elav=: embryonic lethal abnormal vision
eIF2alpha =: eukaryotic initiation factor 2 alpha
ELISA =: enzyme-linked immunosorbent assay
ER =: endoplasmic reticulum
FACS =: fluorescence-activated cell sorting
GFP =: green fluorescent protein
GO =: gene ontology
GS =: gene-switch
Hif1 =: hypoxia-inducible factor 1
Ldh =: lactate dehydrogenase
NADH =: nicotinamide adenine dinucleotide
PERK =: protein kinase R-like endoplasmic reticulum kinase
qRTPCR =: quantitative reverse transcription polymerase chain reaction
RNAi =: RNA interference
RU =: Mifepristone
sima =: similar
SYA =: sugar-yeast-agar
TCA =: trichloroacetic acid
UAS =: upstream activating sequence
UPR =: unfolded protein response
