# Supplementary material for: Activating transcription factor 4-dependent lactate dehydrogenase activation as a protective response to amyloid beta toxicity
Source: Brain Commun. 2021 Mar 26;3(2):fcab053. doi: 10.1093/braincomms/fcab053 (PMC8093921; doi:10.1093/braincomms/fcab053)
Supplement: fcab053_Supplementary_Data [file fcab053_supplementary_data.zip › Supplementary_material.pdf]

SUPPLEMENTARY TABLES:

Supplementary Table 1. Differentially expressed genes in *Drosophila* brain following Aβ42 induction at 5 days of age.
Differential gene expression data analysis (see methods) in brains of 19-day-old flies expressing human Aβ42 peptide from 5 days of age compared to healthy controls.

Supplementary Table 2. Differentially expressed genes in *Drosophila* brain following Aβ42 induction at 20 days of age.
Differential gene expression data analysis (see methods) in brains of 34-day-old flies expressing human Aβ42 peptide from 20 days of age compared to healthy controls.

Supplementary Table 3. Overlapping differentially-expressed genes in fly brain following Aβ42 induction in young versus older ages
Summary of overlapping differentially-expressed genes in response to Aβ42 in both young and old flies (see Fig 1 A).

Supplementary Table 4. Upregulated pathways in *Drosophila* brain following Aβ42 induction at 5 days of age.
Gene Ontology analyses (see methods) depicting upregulated GO terms, and the ranks of genes contained within each category, in brains of 19-day-old flies expressing human Aβ42 peptide from 5 days of age compared to healthy controls.

Supplementary Table 5. Downregulated pathways in *Drosophila* brain following Aβ42 induction at 5 days of age.
Gene Ontology analyses (see methods) depicting downregulated GO terms, and the ranks of genes contained within each category, in brains of 19-day-old flies expressing human Aβ42 peptide from 5 days of age compared to healthy controls.

Supplementary Table 6. Upregulated pathways in *Drosophila* brain following Aβ42 induction at 20 days of age.
Gene Ontology analyses (see methods) depicting upregulated GO terms, and the ranks of genes contained within each category, in brains of 34-day-old flies expressing human Aβ42 peptide from 20 days of age compared to healthy controls.

Supplementary Table 7. Downregulated pathways in *Drosophila* brain following Aβ42 induction at 20 days of age.
Gene Ontology analyses (see methods) depicting downregulated GO terms, and the ranks of genes contained within each category, in brains of 34-day-old flies expressing human Aβ42 peptide from 20 days of age compared to healthy controls.

Supplementary Table 8. Shared candidate genes between *Drosophila* and human cell types
We compared our candidate genes to genes differentially expressed between "no" and "early" Alzheimer's pathology in six cell types from Mathys et al. (2019). Candidate gene sharing between our study and each of the cell types was based on *Drosophila* orthologs (see Methods). We used *SuperExactTest in R* to determine if the number of overlapping genes is expected by chance (sheet 'SuperExactTest Output'). Genes intersecting between flies of 19 or 34 days of age and human data are shown in the sheets 'Genes in Overlap 19d' and 'Genes in Overlap 34d'. Human cell types are

abbreviated as: Ex, excitatory neurons; In, inhibitory neurons; Ast, astrocytes; Oli, oligodendrocytes; Opc, oligodendrocyte progenitor cells; Mic, microglia. Biological process gene ontology annotations for *Drosophila* genes were obtained from FlyBase (March 3, 2020).

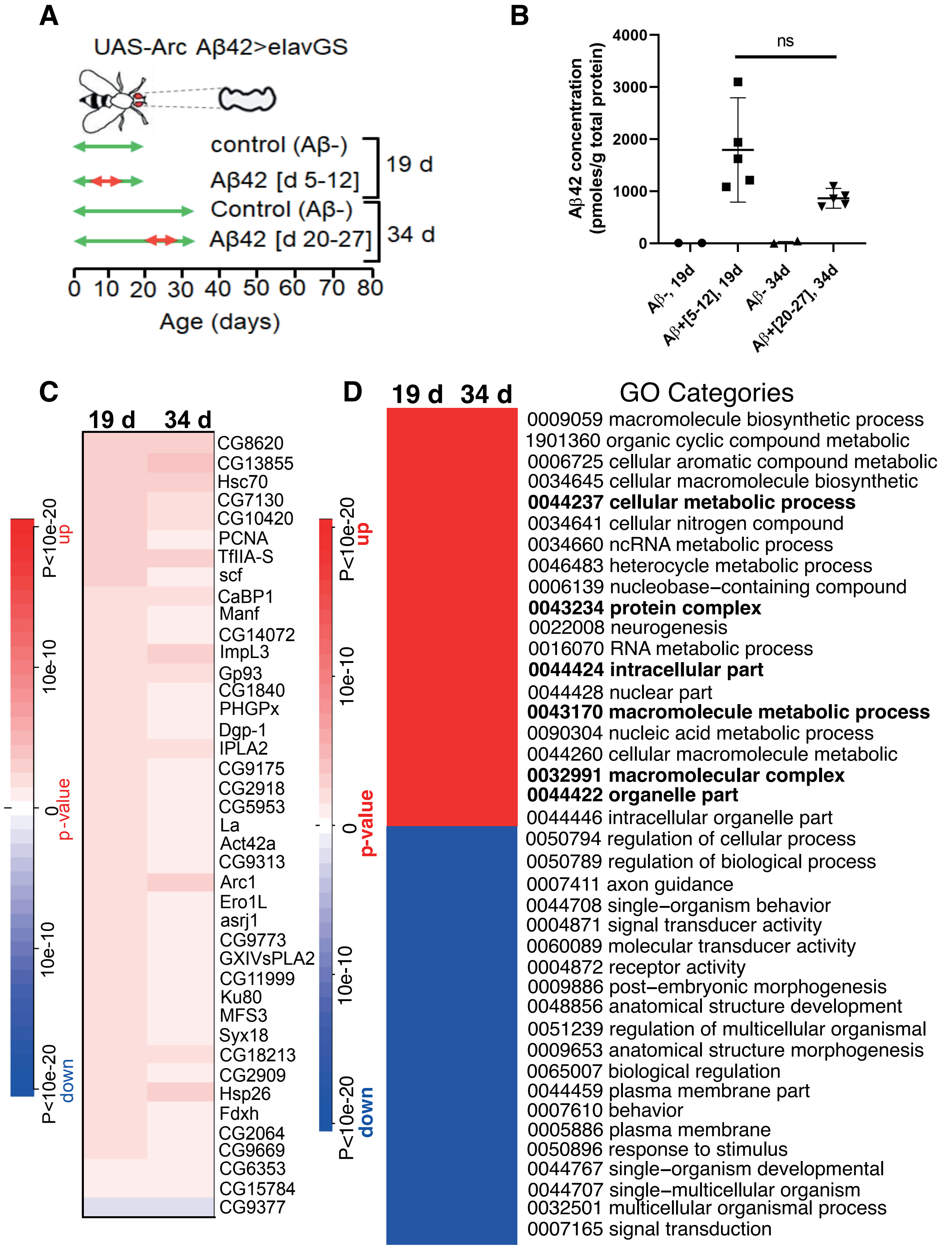

Supplementary Figure 1

A. Flies expressing UAS-ArcAß42, under the control of an RU486-inducible neuronal driver (elavGS), were induced for 1 week at either 5 or 20 days of age . Flies were aged for a further 1 week (14d following induction), then brains dissected for microarray analyses at 19d vs 34d (n=5, 25 brains per biological repeat).

B. Aß42 peptide levels were measured in heads of flies, sampled from the induction described in A (n=4-5, 20 heads per biological replicate). Data were not normally distributed (Shapiro-Wilk) and no significant differences in overall Aß42 peptide were observed between age-groups (p>0.999 comparing Aß+ 19d vs Aß+ 34d; Kruskal-Wallis test followed by Dunn's multiple comparisons).

C. Heat-map depicting 41 commonly differentially expressed genes in young (19 day) and old (34 day) Aß42-expressing vs control fly brains. Red represents increased expression and blue reduced expression (scale = log10 fold change).

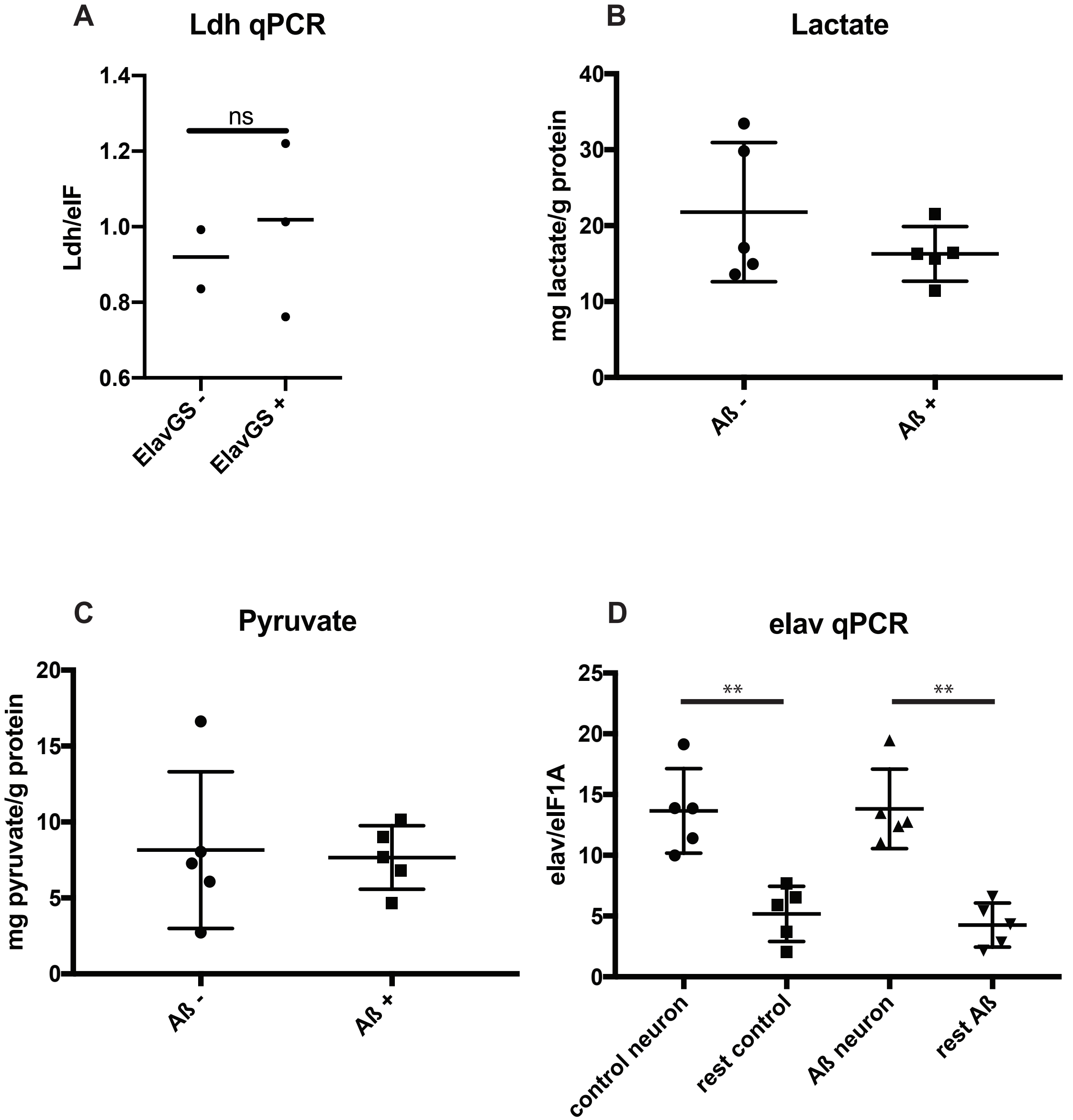

Supplementary Figure 2. A. Ldh qPCR on elavGS flies with (+) and without RU (-) (N=3). B. Lactate levels in Aß (Aß+) and control (Aß-) expressing brains (N=3). C. Pyruvate levels in Aß (Aß+) and control (Aß-) expressing brains (N=3). D. elav qPCR analysis of FACS sorted GFP expressing neurons and other cells, expressing Aß (Aß) and driver alone (controls) (N=5). This shows that FACS sorted GFP cells were mostly neurons. Genotypes: (A) *elavGS.* (B) *UAS-Aß;elavGS*. (C) *UAS-Aß/UAS-eG-FP; elav GS and UAS-eGFP; elav GS.* Comparisons were carried out by student's t-test or, for FACS-sorted neurons, one-way ANOVA and Tukey's post-hoc comparisons. ** p<0.01

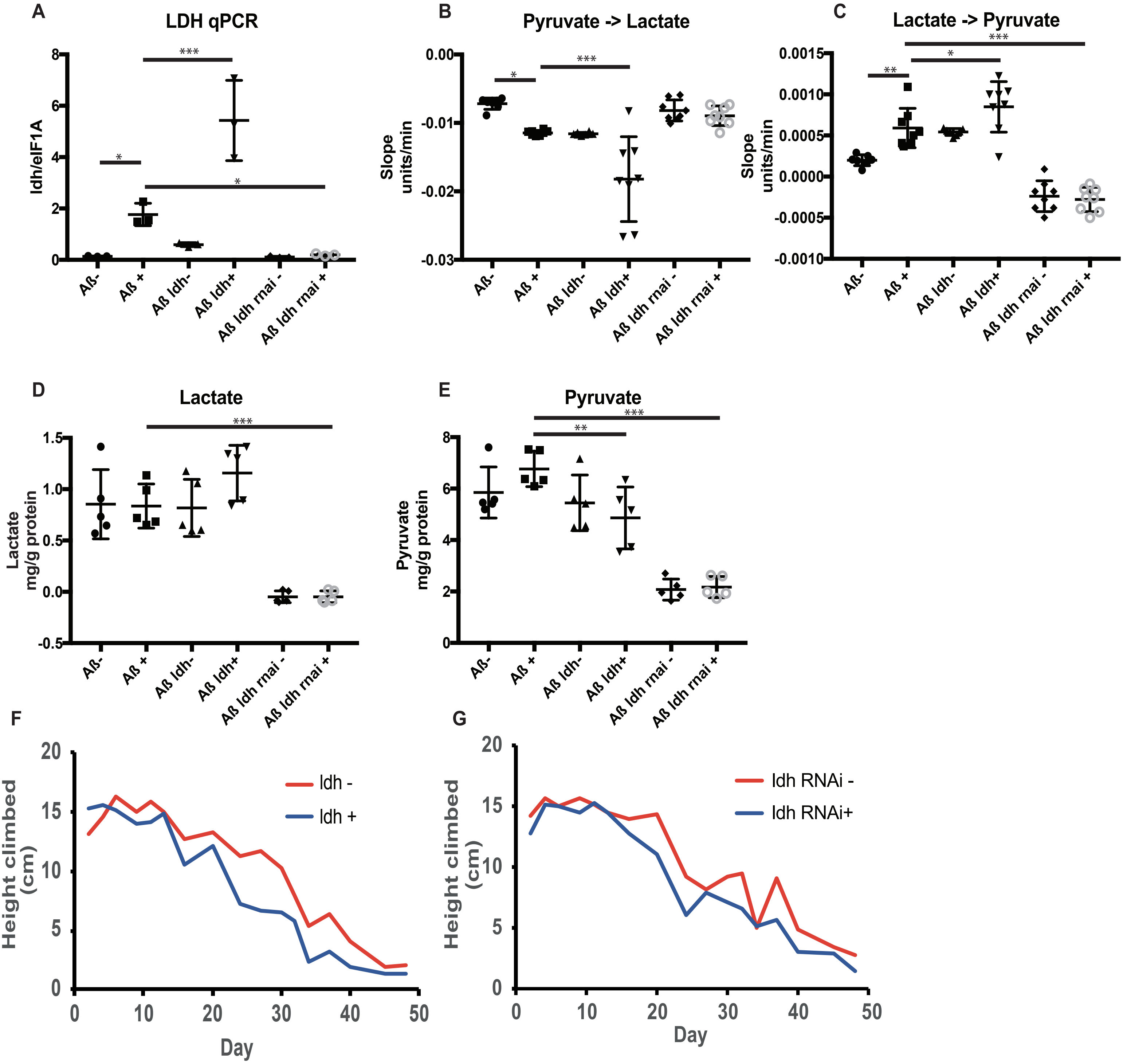

Supplementary Figure 3. Characterisation of RNAi and over-expression line. A. Ldh qPCR analysis of brains expressing Aß, Aß+Ldh and Aß+LdhRNAi (+) and uninduced controls (Aß-). (N=3, per condition). B, C Ldh enzymatic assay on brain extracts expressing Aß, Aß+Ldh and Aß+LdhRNAi (+) and uninduced controls (-) (N=7-8 per condition). Assayed in the direction of lactate production (B) and pyruvate production (C). Values shown are the slopes generated by the enzymatic reaction. Lactate production generates a negative slope, so a lower negative value signifies a greater activity. D. Lactate levels in Aß, Aß+Ldh and Aß+LdhRNAi (+) expressing brains and control (-). E. Pyruvate levels in Aß, Aß+Ldh and Aß +LdhRNAi (+) expressing brains and control (-). Genotypes: *UAS-Aß/UAS-Ldh; elav GS, UAS-Aß;elavGS. UAS-Aß/UAS-Ldh RNAi; elav GS, UAS-Aß;elavGS* (N=5, for lactate and pyruvate). Comparisons were carried by ANOVA followed by Sidak's multiple comparison test. * $p<0.05$ ** $p<0.01$, *** $p<0.005$. F. Climbing assay of flies expressing Ldh in the brain of otherwise wild type flies. Expression of Ldh significantly reduces climbing during ageing (p=3.13e-12 by linear regression for interaction of RU with time) Genotype: *UAS-Ldh; elavGS*. (n>100 per condition) G. Climbing assay of flies expressing LdhRNAi in the brain of otherwise wild type flies. Downregulation of Ldh significantly reduces climbing during ageing (p= 0.02419 by linear regression for interaction of RU with time) Genotype: *UAS-Ldh; elavGS* (n>100 per condition).

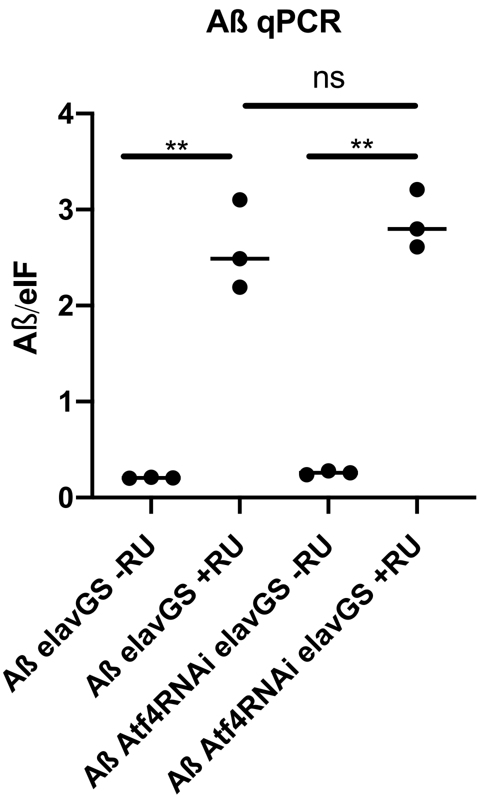

Supplementary Figure 4. Aß mRNA levels measured by qPCR in Aß, Aß+Atf4RNAi (+) and uninduced controls (-), showing that co-overexpression of Atf4RNAi does not affect Aß levels.
Genotypes: *UAS-Aß/UAS-Atf4 RNAi; elavGS, UAS-Aß;elavGS* (N=3 per condition). Comparisons were carried by ANOVA followed by Tukey's multiple comparison test. ** p<0.01
